# Anatomy of the Ileum, Intestinal Glands and Spleen

**Published:** 1911-07

**Authors:** J. F. Grant

**Affiliations:** Prof. of Anatomy, Atlanta College of Physicians and Surgeons, Atlanta


### Journal-Record of Medicine

Successor to Atlanta Medical and Surgical Journal, Established 1855, and Southern Medical Record, Established 1870.

**OWNED BY THE ATLANTA MEDICAL JOURNAL CO.**

**Published Monthly**

*Official Organ Fulton County Medical Society, State Examining Board, Presbyterian Hospital, Atlanta, Birmingham and Atlantic Railroad Surgeons' Association, Chattahoochee Valley Medical and Surgical Association, Etc.*

EDGAR G. BALLENGER., M. D., Editor.
BERNARD WOLFF, M. D., Supervising Editor.
A. W. STIRLING, M. D., C. M., D. P. H., J. S. HURT, B. Ph., M. D.
GEO. M. NILES, M. D., W. J. LOVE, M. D., (Ala.); Associate Editors.
E. W. ALLEN, Business Manager.

COLLABORATORS

Dr. W. F. WESTMORLAND, General Surgery.
F. W. McRAE, M. D., Abdominal Surgery.
H. F. HARRIS, M. D., Pathology and Bacteriology.
E. B. BLOCK, M. D., Diseases of the Nervous System.
MICHAEL HOKE, M. D., Orthopedic Surgery.
CYRUS W. STRICKLER, M. D., Legal Medicine and Medical Legislation.
E. C. DAVIS, A. B., M. D., Obstetrics.
E. G. JONES, A. B., M. D., Gynecology.
R. T. DORSEY, Jr., B. S. M. D., Medicine.
L. M. GAINES, A. B., M. D., Internal Medicine.
J. N. LeCONTE, M. D., Disease of the Stomach and Intestines.
L. B. CLARKE, M. D., Pediatrics.
EDGAR PAULIN, M. D., Opsonic Medicine.
THEODORE TOEPEL, M. D., Mechano Therapy.
R. R. DALY, M. D., Medical Society.
A. W. STIRLING, M. D., etc., Diseases of the Eye, Ear, Nose and Throat.
BERNARD WOLFF, M. D., Diseases of the Skin.
E. G. BALLENGER, M. D., Diseases of the Genito-Urinary Organs.

VOL. LVIII. JULY, 1911 NO. 4.

### SYMPOSIUM ON TYPHOID FEVER

### ANATOMY OF THE ILEUM, INTESTINAL GLANDS AND SPLEEN.

BY DR. J. F. GRANT, PH.B., M.D., PROF. OF ANATOMY, ATLANTA COLLEGE OF PHYSICIANS AND SURGEONS, ATLANTA.

The ileum, according to the more usual descriptions, comprises the lower three-fifths of the small intestine below the duodenum. Closely similar in structure and arrangement to the upper portion of the jejuno-ileum, this part of the gut is frequently found in characteristic coils in the right lower quadrant of the abdominal cavity and is normally separated from the anterior abdominal wall by the great omentum. It is suspended by a double layer of peritoneum, the necentery proper, which secures the ileum to the dorsal abdominal wall and is scarcely more than six inches in length in the sagittal direction, extending obliquely from the left side of the first or second lumbar vertebra to the right sacro-iliac synchondrosis. The dorso-ventral length of the mesentery is generally about six inches, but the maximum length of mesentery attaches that portion of the bowel included between a point six feet from the duodenum and one eleven feet from the same point. The mesentery here attains a length of ten inches. This part of the ileum enjoys the greatest mobility, and Treves has pointed out that this portion, which includes the commencement of the ileum, is the most dependent and usually lies, partly in the pelvic cavity.

*Histology.* The four tunics of the intestinal wall include the mucosa, submucosa, muscularis and serosa. Mall has described the *mucosa* as being primarily made up of a connective tissue reticulum bearing numerous lymphoid cells in its meshes. The development of epithelial glandular structures displaces to a large extent this stroma so that it becomes reduced in amount in proportion to the epithelial development in this tunic, and remains as a supporting structure for the epithelial formations. This point has some significance in our present consideration owing to the facility which this loose tissue offers for invasion by nicroorganisms. The surface is everywhere clothed with a simple columnar epithelium, exhibiting a striated appearance along tthe margin. Villi propect above the general surface-level and increase considerably the surface area. Their interior is formed of mucosal stroma and contains numerous smooth muscle cells derived from the muscularis mucosae. Between the bases of adjacent villi simple tubular glands open upon the surface, their epithelium being directly continuous with that covering the surface. Goblet cells with mucous secretion are plentiful both on the surface and in the tubular glands, and leucocytes are numerous between the cells of the surface epithelium. The mucosa enjoys considerable mobility upon its loosely constructed *submucosa,* formed of lax connective tissue bearing large blood-vessels and lymphatics. The

*muscularis,* disposed in two layers of smooth muscle, lies between the submucosa and the firm connective tissue of the *serosa,* the latter being covered with peritoneum. Permanent circular folds or Valvulac conniventes of the submucosa slightly encroach upon the lumen of the bowel covered with the entire structure of the mucosa. By their transverse disposition in the lumen the intestinal contents may be partially impeded in their onward progression, and digestion and absorption thereby facilitated. Large numbers of *simple follicles* of lymphoid tissue are scattered throughout the mucosa of the ileum, particularly toward the lower extremity, the larger ones penetrating into the submucosa. Each one has a pale interior, the "germ centre," where cell reproduction is in active progress. Of greater interest in our present study are the *Peyer's patches,* consisting of closely-packed aggregations of simple follicles, occupying both mucosa and submucosa. These ordinarily number about thirty, and may be scattered throughout the length of the ileum. Peyer's areas are always situated opposite the mesenteric attachment and are frequently more numerous toward the caecal extremity where the most extensive intestinal changes in typhoid are usually observed.

The *lymphatics* of the ileum being as slender radicles supported by the reticular and lymphoid stroma in the core of the villi and proceed to the submucosa where the large lymphatics are found, thence, issuing through the outer coats to the lymph vessels in the mesentery. Here are interposed along their course seevral groups of lymph nodes, the so-called mesenteric glands. The largest of these are found close to the origin of the mesentery and the mesenteric artery. These further communicate with the retroperitoneal glands in relation with ththe aorta. The onward flow if lymph continues through the receptaculum chyli and the thoracic duct. The *arterial supply* is exclusively through the superior meseneric artery which enters the mesenery at its origin and proceeds directly toward the bowel. From the main artery branches arise which anastomose with adjacent branches to form loops (primary loops). From these loops smaller and more numerous branches spring which unite to form secondary loops. In the same manner other series of loops may be formed, each successive anastomosis being nearer the bowel. From the last series of loops straight branches or vasa recta springs which pierce the outer tunics of the ileum to gain at once the submucosa, whence subdivisions are distributed to the mucosa internally and the muscularis and serosa externally. In the mucosa the finer arteries form close networks about the tubular glands and underneath the surface epithelium which clothes the villi. The large size of vessels contained in the submucosa is worthy of emphasis since their erosion in deep ulceration through an adenoid patch may occasion profuse hemorrhage. The *discharge of venous blood* from the ileum is through the portal system and originates as small vessels within the villi and about the tubular glands. Usually only one proceeds from a single villus, the small adjacent vessels and astomosing freely in the mucosa and submucosa and forming in the latter large venous trunks which pass in company with the corresponding arteries to the main vessels in the mesentery. The course of this vein toward the liver and the presence of considerable-sized perivascular lymphatic spaces about its wall are facts of considerable import in effecting the dissemination of toxic and of septic materials once they have passed the limits of the intestinal walls, and the interposed lymph-nodes. The *nerves* of the bowel are both medullated and non-medullated fibres derive from the central and sympathetic systems. The association of the lower thoracic spinal nerves with those which are destined to supply the ileum may be of interest when we consider their relation to the nerves supplying the abdominal muscles and skin. An explanation is thereby offered as to the reflex machanism involved in the production of abdominal tenderness, rigidity of the abdominal muscles, meteorism (as possibly due to vaso-motor disturbances), and the referred pains experienced in the umbilical region or epigastrium in the early stage of peritoneal involvement.

*Topography.* The location of the various parts of the convoluted intestine has been the subject of investigation by several workers, notably Mall, who traced the changes in position of the various parts during the developmental changes from embroyo to adult, and Sir Frederick Treves who examined adult bodies. The results of their investigations have established only the relation of the parts and has not established criteria by which any definite part may be identified from surface landmarks, or with reference to adjacent coils. The ileum is mainly located in the hypogastric and right iliac regions. The coils of the left side are commonly transversely arranged, those on the right of the median line being generally vertical, the terminal part of the ileum emerging from the pelvis to join the caecum in the right iliac region. Normal variations from this disposition of the coils is to be expected from the great mobility of the organ. The ileo-caecal junction has of course a fairly fixed location, and the ileum may be conveniently traced upward from this point. Other factors of relative importance in identifying this part of the tract the (1) the diameter of the intestine, which becomes progressively smaller from above downwards; (2) The thickness of the muscular coat which is greater above than below; (3) Valvulae conniventes which may be observed through the intact bowel, are prominent in the duodenal extremity and become progressively less developed toward the caecum. They are small and scanty in the ileum and in the lower third are frequently wanting;. (4) The arterial loops are primary in the upper part of the intestine and the vasa recta long, from one to two inches. Toward the lower extremity there are tertiary loops or as many as four or five series of loops, and the vasa recta are short, one-half to one inch.

The attachment of the mesentery has already been mentioned. The continuity of the mesentery in a sagital direction tends to prevent the passage of small amounts of extravasated fluids from one side to the other. It is of surgical significance to note that extravasation of intestinal contents or of blood in the peritoneal cavity on the right side of the mesentery is usually directed into the right iliac fossa, and on the left side it is commonly discovered in the pelvis.

### THE SPLEEN.

This organ lies in the left hypochrondrium and to a slight extent in the epigastrium. Its position is determined largely by two factors: (1) its ligaments and their condition of normal tension or abnormal laxity, and (2) the condition of the mobile organs with which it is in relation. Its ligaments are peritoneal folds and include the gastro-splenic omentum, the lieno-renal ligament and the suspensory ligamentt. Its related organs are the stomach, the lung, from which it is separated by the diaphragm, the eplenic flexure of the colon, the flidney or peritoneum, the lesser sac approaching the hilum. It is located under the ninth, tenth and eleventh ribs, its long axis corresponding with the tenth. It may be palpated only when displaced or enlarged in which latter condition it may be felt under the tip of the tenths rib.

Histologically, the spleen is composed of lymphoid tissue and larger splenic pulp cells supported on a reticular tissue, the coarser framework being composed of broad interlacing bands of fibrous and smooth muscle tissues. The large and tortuous splenic artery enters the organ at its hilum and cources through the fibrous trabeculae, sending off smaller branches which penetrate the pulp, become surrounded by dense aggregations of lymphoid cells, the Malpighian corpuscles, and ultimately being distributed through minute branches which end abruptly in the pump. A break or gap appears to exist between the arterioles and the smallest venules where the blood is in intimate relation with the tissue of the organ. The veins begin as funnel-shaped radicles which unite to form larger vessels approaching the trabeculoe and ultimately make their exit through the hilum as the splenic vein which is the largest constituont of the portal. The lack of anastomosis between the arteries in the spleen is a notegan are directed toward the ileum and accompany the vessels. Splenic lymph glands are found along the course of the different lymphatic vessels. The nerves are derived from the splenic plexus of the sympathetic.

